# Data of ^1^H/^13^C NMR spectra and degree of substitution for chitosan alkyl urea

**DOI:** 10.1016/j.dib.2016.03.099

**Published:** 2016-04-04

**Authors:** Jing Wang, Ji-Zhou Jiang, Wei Chen, Zheng-Wu Bai

**Affiliations:** a School of Chemistry and Environmental Engineering, Wuhan Institute of Technology, Wuhan 430073, PR China; b Department of Physics, National University of Singapore, 2 Science Drive 3, Singapore 117542, Singapore

## Abstract

The data shown in this article are related to the subject of an article in Carbohydrate Polymers, entitled “Synthesis and characterization of chitosan alkyl urea” [1]. ^1^H NMR and ^13^C NMR spectra of chitosan *n*-octyl urea, chitosan *n*-dodecyl urea and chitosan cyclohexyl urea are displayed. The chemical shifts of proton and carbon of glucose skeleton in these chitosan derivatives are designated in detail. Besides, ^1^H NMR spectra of chitosan cyclopropyl urea, chitosan *tert*-butyl urea, chitosan phenyl urea and chitosan *N*,*N*-diethyl urea and the estimation of the degree of substitution are also presented. The corresponding explanations can be found in the above-mentioned article.

## **Specifications Table**

**Table:** 

Subject area	Chemistry
More specific subject area	Polysaccharides modification
Type of data	Table, text file, graph, figure
How data was acquired	High-resolution liquid NMR 600 MHz spectrometer of Bruker Avance III (Sweden) with a 5 mm TCI CryoProbe equipped with Z-gradients up to 53 G/cm, and NMR 400 MHz spectrometer of Varian (USA)
Data format	Analyzed
Experimental factors	Sample solutions (20 mg/ml) were prepared with deuterated trifluoroacetic acid (TFA-D) as solvent. TFA-D was also employed as reference: *δ* 11.50 ppm for proton and *δ* 164.1 ppm for carbon
Experimental features	Detection temperature was set at 25 °C. Samples were scanned 64 times for ^1^H NMR spectra measurement, and scanned 1 h for ^13^C NMR measurement
Data source location	Qingdao, PR China; Wuhan, PR China
Data accessibility	Data is provided in this article

## Value of the data

• Provide reference data of chemical shifts in ^1^H NMR and ^13^C NMR spectra for related chitosan derivatives.
• The data showed in ^1^H NMR spectra suggest a facile way to estimate degree of substitution of a substituent.
• The chemical shifts designation is helpful to structural analysis of polysaccharides or their derivatives.

## 1 Data

Data presented here is divided into two classes. In the first class, ^1^H NMR and ^13^C NMR spectra of three chitosan alkyl urea derivatives with very high degree of substitution (*DS*) are displayed, and the corresponding chemical shifts of proton and carbon of glucose skeleton in the chitosan derivatives are tabulated. In the second class, ^1^H NMR spectra of chitosan alkyl urea with relatively lower *DS* are showed. According to the integrals in ^1^H NMR spectra, the estimation of every *DS* is described.

## 2 Experimental design, materials and methods

**Fig. 1:**
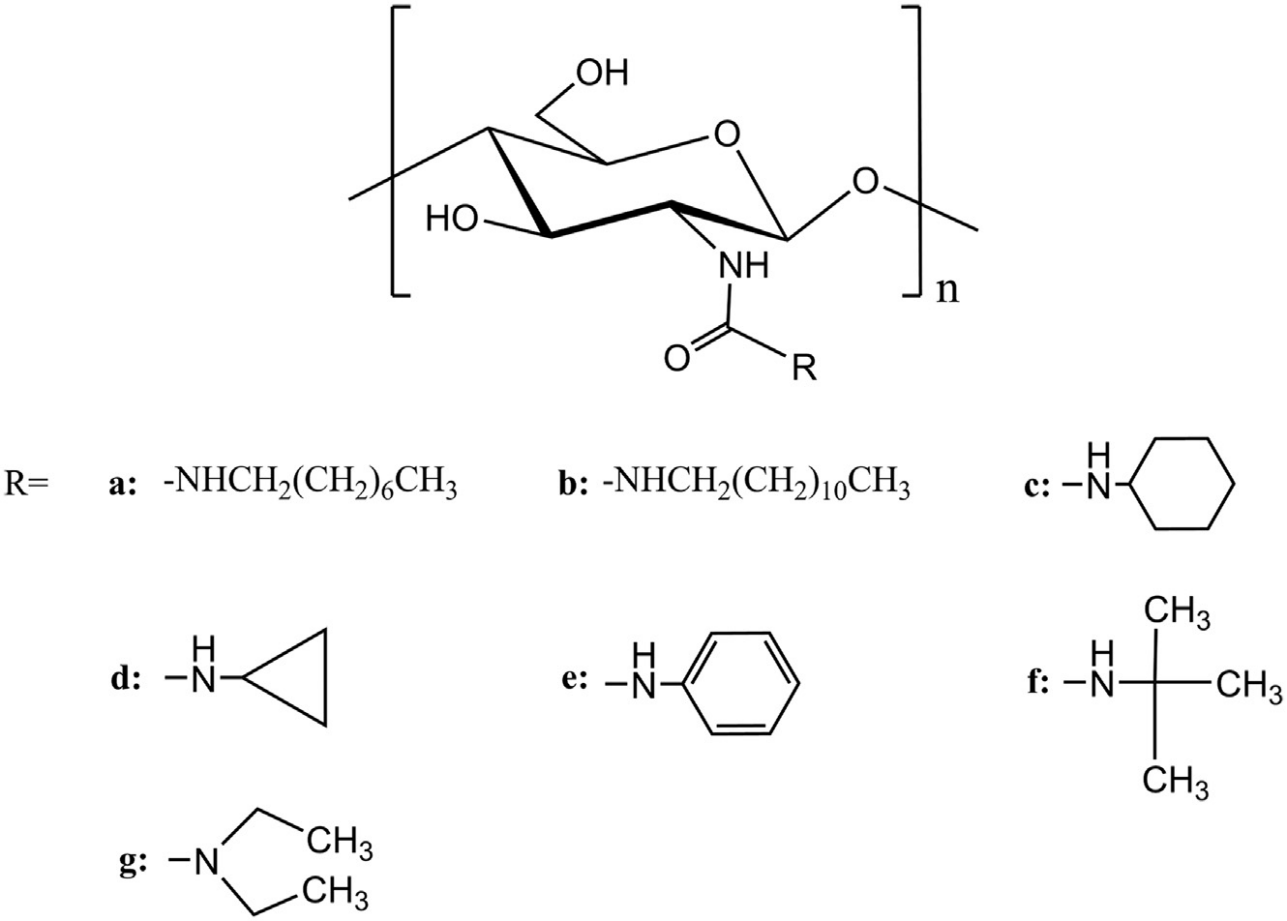
Structures of chitosan alkyl urea derivatives (**a**–**g**).

All chitosan alkyl urea derivatives (Fig. 1) were synthesized with the same method described in Ref. [1]. Specifically, chitosan firstly was reacted with methyl chloroformate yielding *N*-methoxyformylated chitosan, which was converted into chitosan alkyl urea via amine–ester exchange reaction with structurally different amines. After the products were washed with ethanol and dried, ^1^H and ^13^C NMR measurements of **a**–**c** were performed on a high-resolution liquid NMR 600 MHz spectrometer of Bruker Avance III (Sweden) with a 5 mm TCI CryoProbe equipped with Z-gradients up to 53 G/cm. ^1^H NMR measurement of **d**–**g** was conducted on a NMR 400 MHz spectrometer of Varian (USA). Sample solutions (20 mg/ml) were prepared with TFA-D as solvent. TFA-D was also used as internal standard: proton (*δ* 11.50 ppm) and carbon (*δ* 164.1 ppm). Detection temperature was set at 25 ^o^C. Degree of substitution (*DS*) of alkyl group (diethylamido group for **g**) on chitosan alkyl urea were determined according to integrals in corresponding ^1^H NMR spectra.

**Fig. 2:**
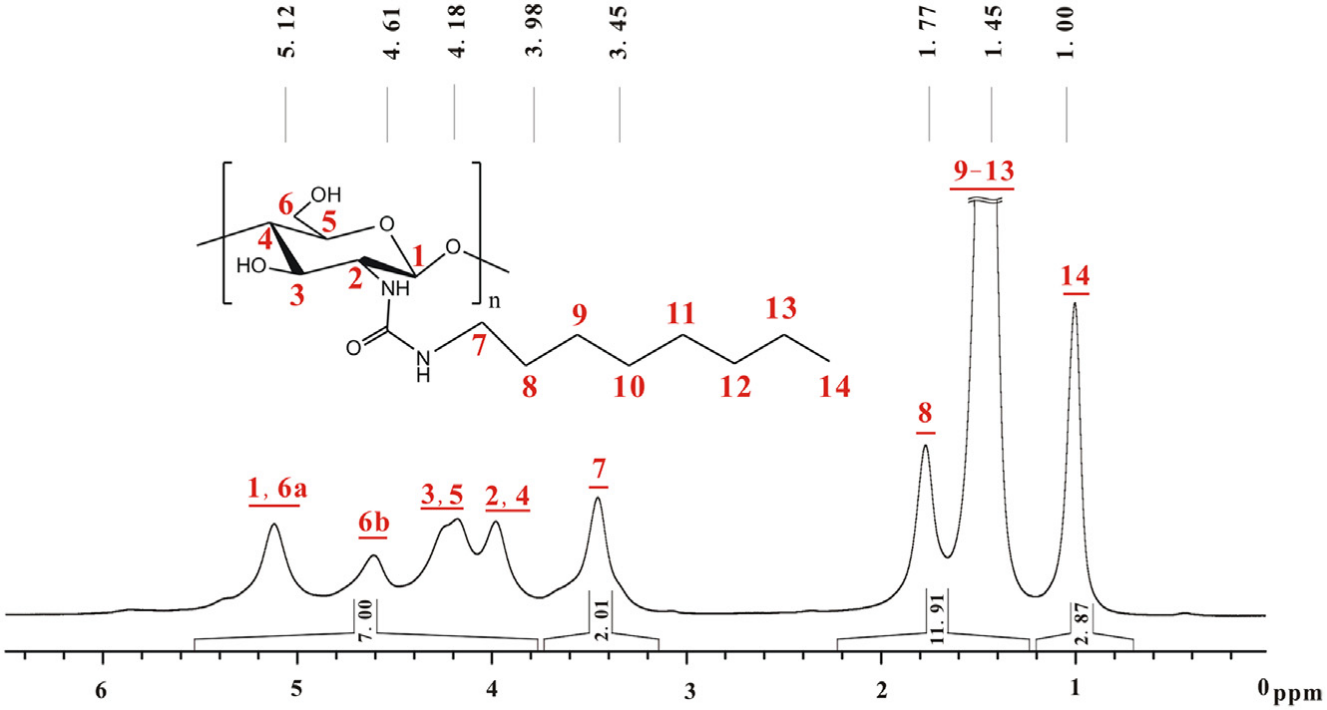
^1^H NMR spectrum of **a** (TFA-D, 298 K, 600 MHz).

As shown in Fig. 2, the peaks from 5.12 to 3.98 ppm correspond to the seven hydrogens of glucose skeleton of chitosan, and their total integral is designated as 7.00. The peak at 3.45 and the peaks from 1.77 to 1.45 ppm theoretically correlate with two hydrogens and twelve hydrogens. The actual integrals almost match the hydrogen numbers, and the integral of the peak at 1.00 is close to 3.00. Therefore, the *DS* of *n*-octyl on **a** is nearly 100%.

**Fig. 3:**
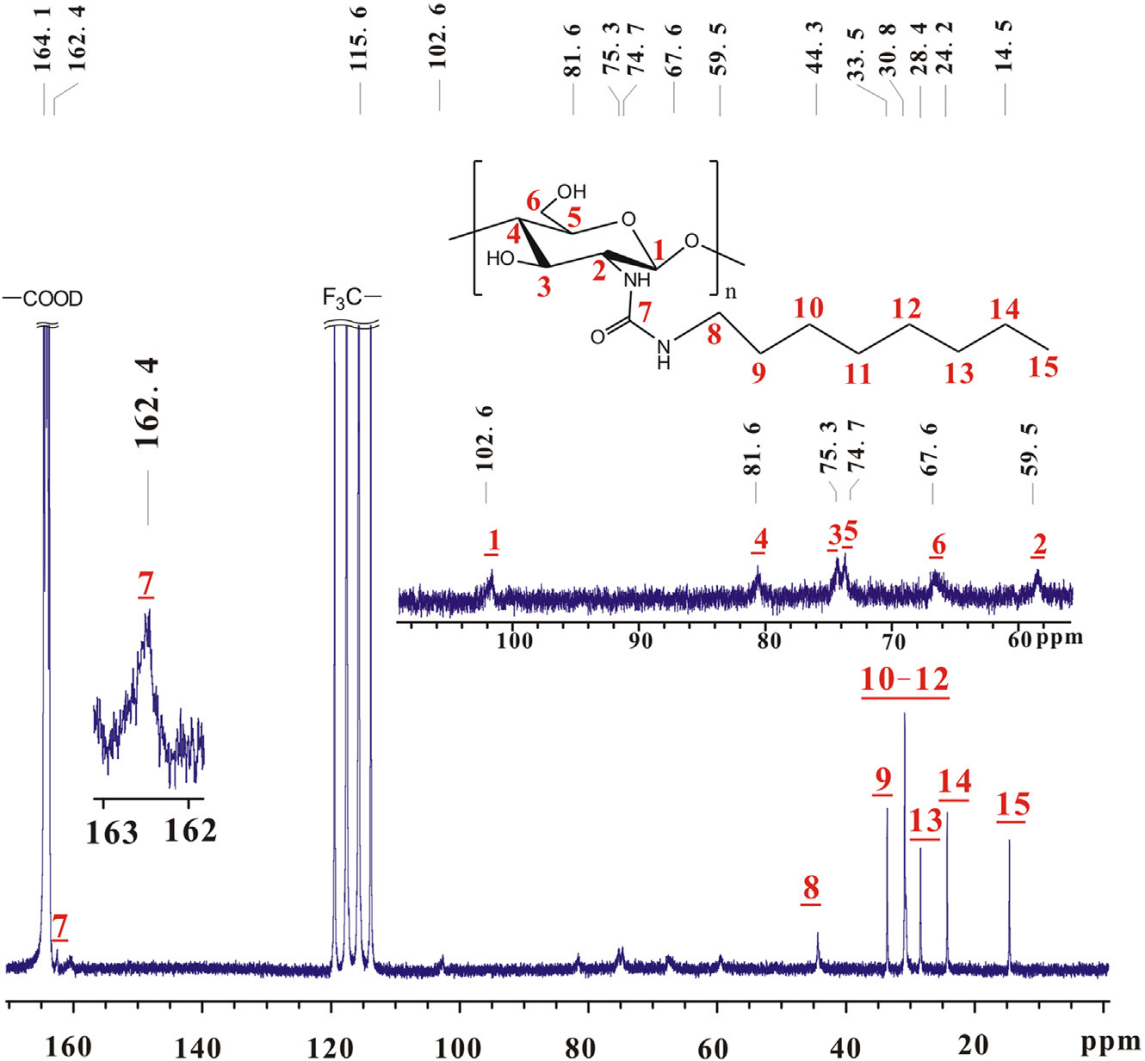
^13^C NMR spectrum of **a** (TFA-D, 298 K, 600 MHz).

Based on Fig. 2, Fig. 3, the chemical shifts of proton and carbon in glucose skeleton of chitosan *n*-octyl urea (**a**) are listed in Table 1. The sequence of chemical shifts of C_1_–C_6_ slightly differs from that in reported [2], [3].

**Table 1:** Chemical shifts of proton and carbon in.glucose skeleton of chitosan *n*-octyl urea (**a**).

	^1^H NMR *δ* (ppm)		^13^C NMR *δ* (ppm)
H_1_	5.12	C_1_	102.6
H_2_	3.98	C_2_	59.5
H_3_	4.26	C_3_	75.3
H_4_	3.98	C_4_	81.6
H_5_	4.18	C_5_	74.7
H_6_	4.61, 5.12	C_6_	67.6

**Fig. 4:**
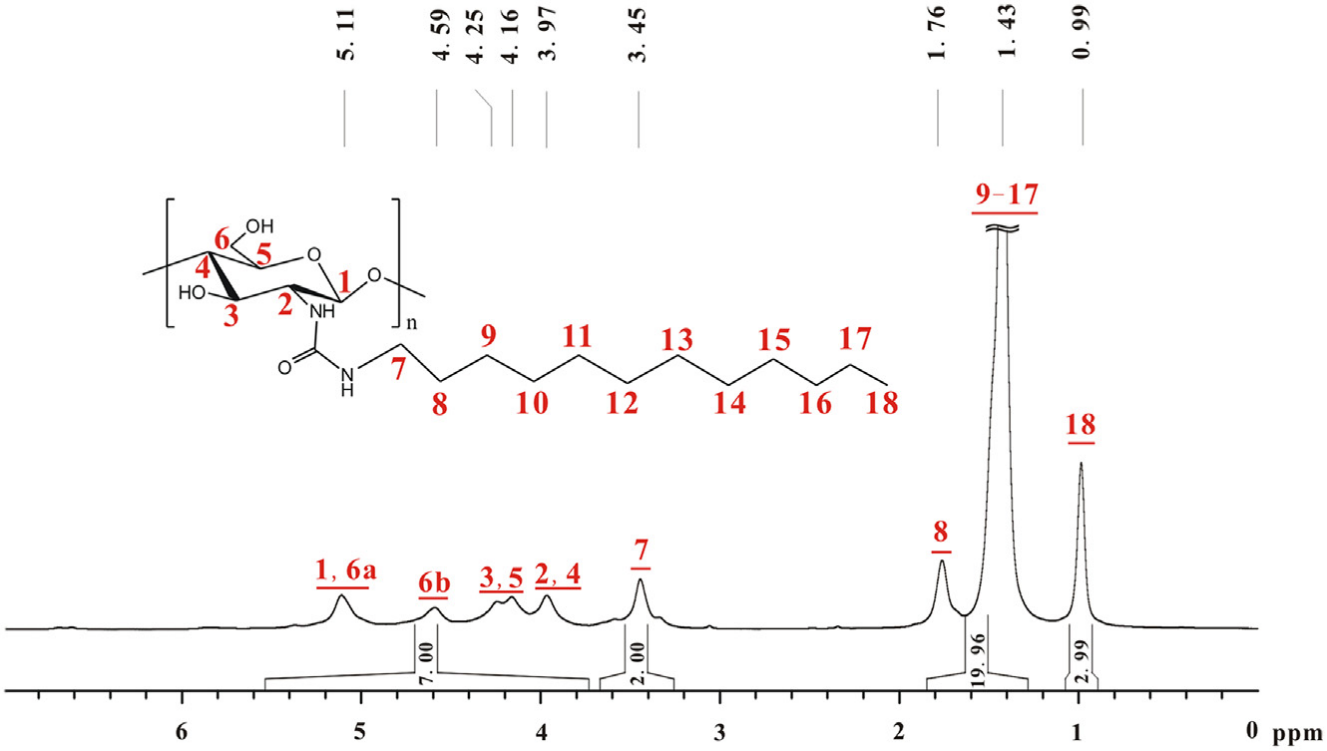
^1^H NMR spectrum of **b** (TFA-D, 298 K, 600 MHz).

The total integral of the seven hydrogens of glucose skeleton of chitosan is designated as 7.00 (Fig. 4), other integrals match corresponding hydrogen numbers. Therefore, the *DS* of *n*-dodecyl on **b** is almost 100%.

**Fig. 5:**
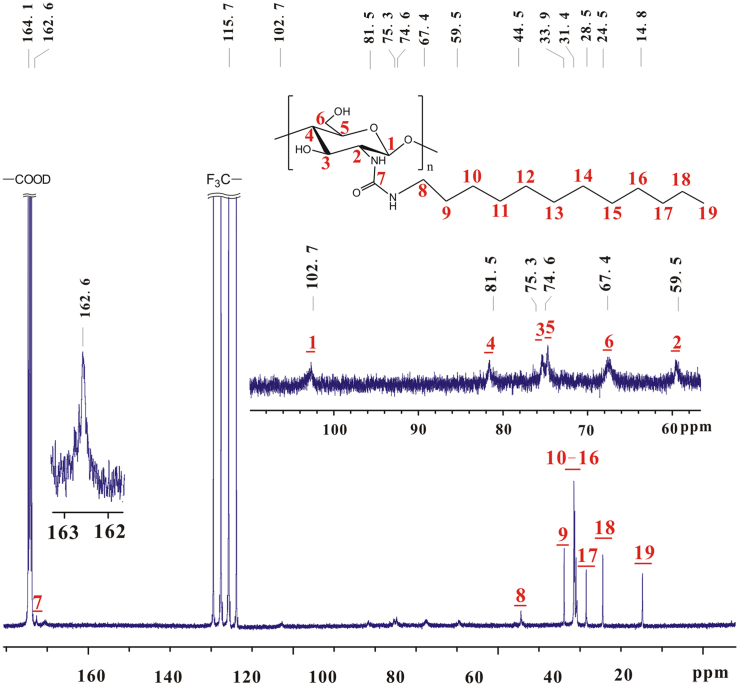
^13^C NMR spectrum of **b** (TFA-D, 298 K, 600 MHz).

Based on Fig. 4, Fig. 5, the chemical shifts of proton and carbon in glucose skeleton of chitosan *n*-dodecyl urea (**b**) are listed in Table 2.

**Table 2:** Chemical shifts of proton and carbon in glucose skeleton of chitosan *n*-octyl urea (**b**).

	^1^H NMR *δ* (ppm)		^13^C NMR *δ* (ppm)
H_1_	5.11	C_1_	102.7
H_2_	3.97	C_2_	59.5
H_3_	4.25	C_3_	75.3
H_4_	3.97	C_4_	81.5
H_5_	4.16	C_5_	74.6
H_6_	4.59, 5.11	C_6_	67.4

**Fig. 6:**
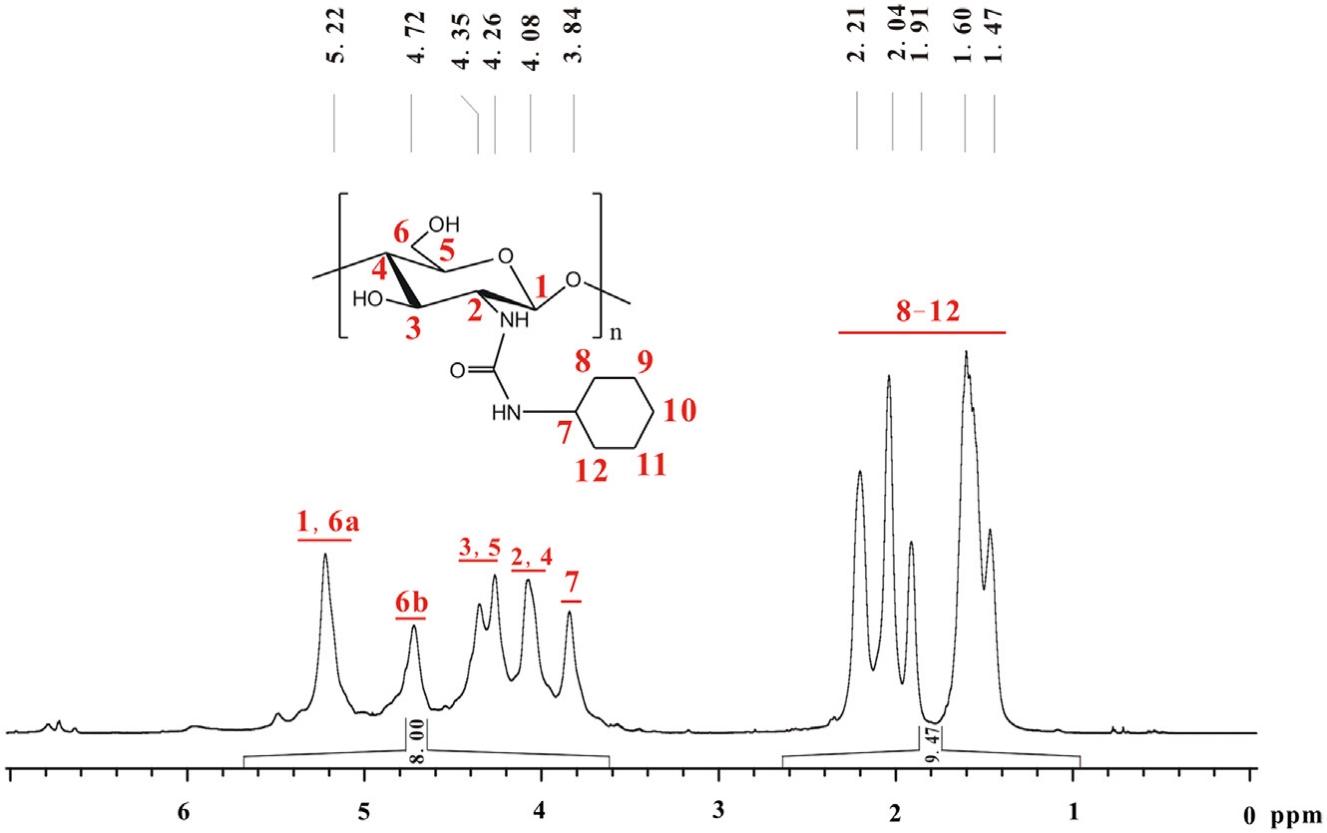
^1^H NMR spectrum of **c** (TFA-D, 298 K, 600 MHz).

The peak of the hydrogen in –CH– of cyclohexyl group overlapped with that of seven hydrogens on glucose skeleton of chitosan, and the total integral of these eight hydrogens is assumed as 8.00 (Fig. 6). The *DS* of cyclohexyl group on **c** is calculated by the equation of (7+*DS*)/10*DS*=8.00/9.47. Therefore, *DS*=94%.

**Fig. 7:**
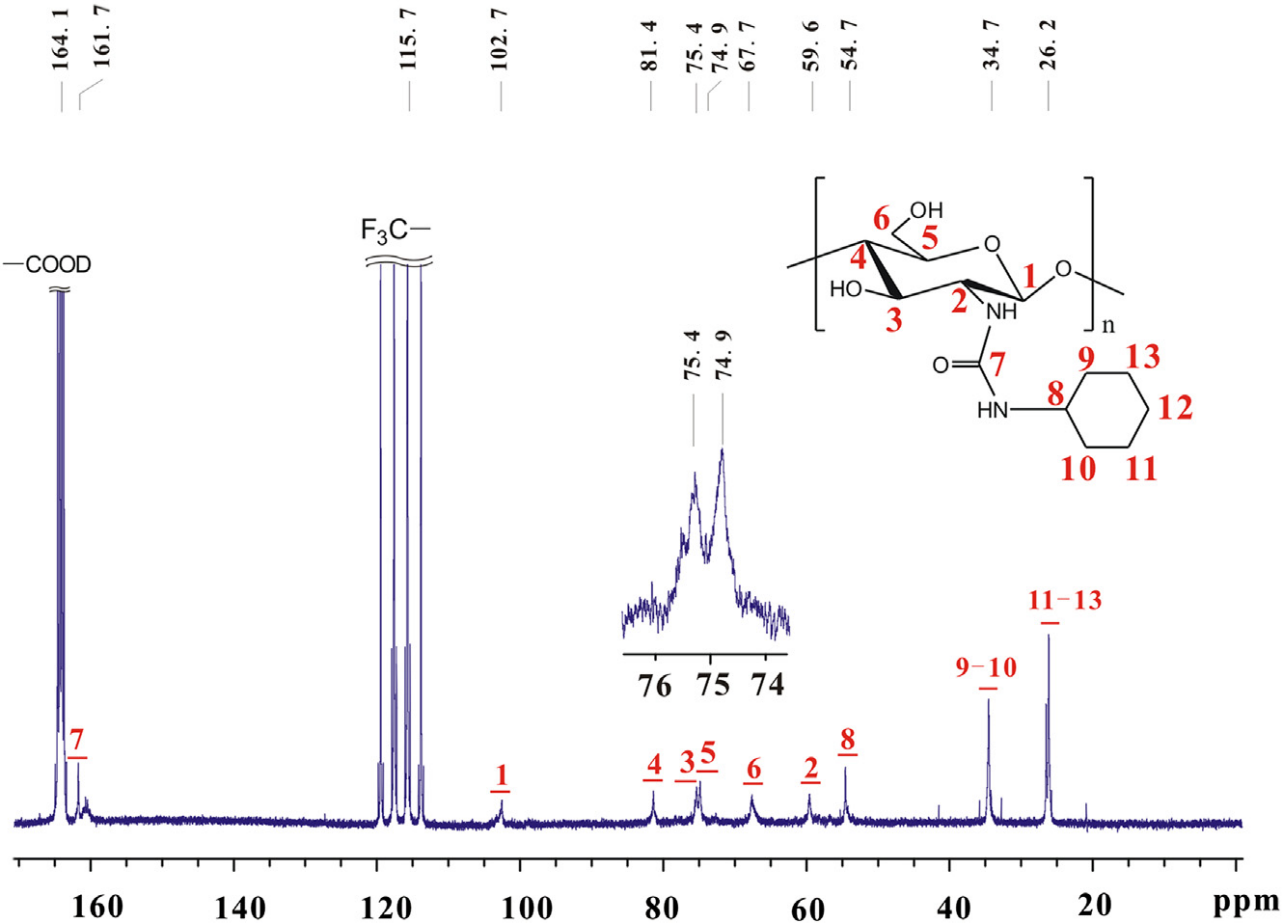
^13^C NMR spectrum of **c** (TFA-D, 298 K, 600 MHz).

Based on Fig. 6, Fig. 7, the chemical shifts of proton and carbon in glucose skeleton of chitosan cyclohexyl urea (**c**) are listed in Table 3.

**Table 3:** Chemical shifts of proton and carbon in glucose skeleton of chitosan cyclohexyl urea (**c**).

	^1^H NMR *δ* (ppm)		^13^C NMR *δ* (ppm)
H_1_	5.22	C_1_	102.7
H_2_	4.08	C_2_	59.6
H_3_	4.35	C_3_	75.4
H_4_	4.08	C_4_	81.4
H_5_	4.26	C_5_	74.9
H_6_	4.72, 5.22	C_6_	67.7

**Fig. 8:**
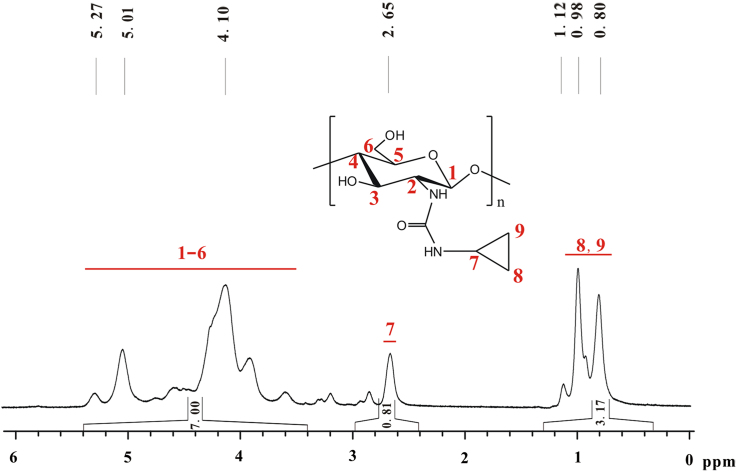
^1^H NMR spectrum of **d** (TFA-D, 298 K, 400 MHz).

The total integral of seven hydrogens on glucose skeleton of chitosan is designated as 7.00 (Fig. 8). The *DS* of cyclopropyl group on **d** equals (*A*/4)×100%. *A* refers to total integral of the peaks from 1.12 to 0.80, in which there are four hydrogens. *DS*=(3.17/4)×100%≈79%.

**Fig. 9:**
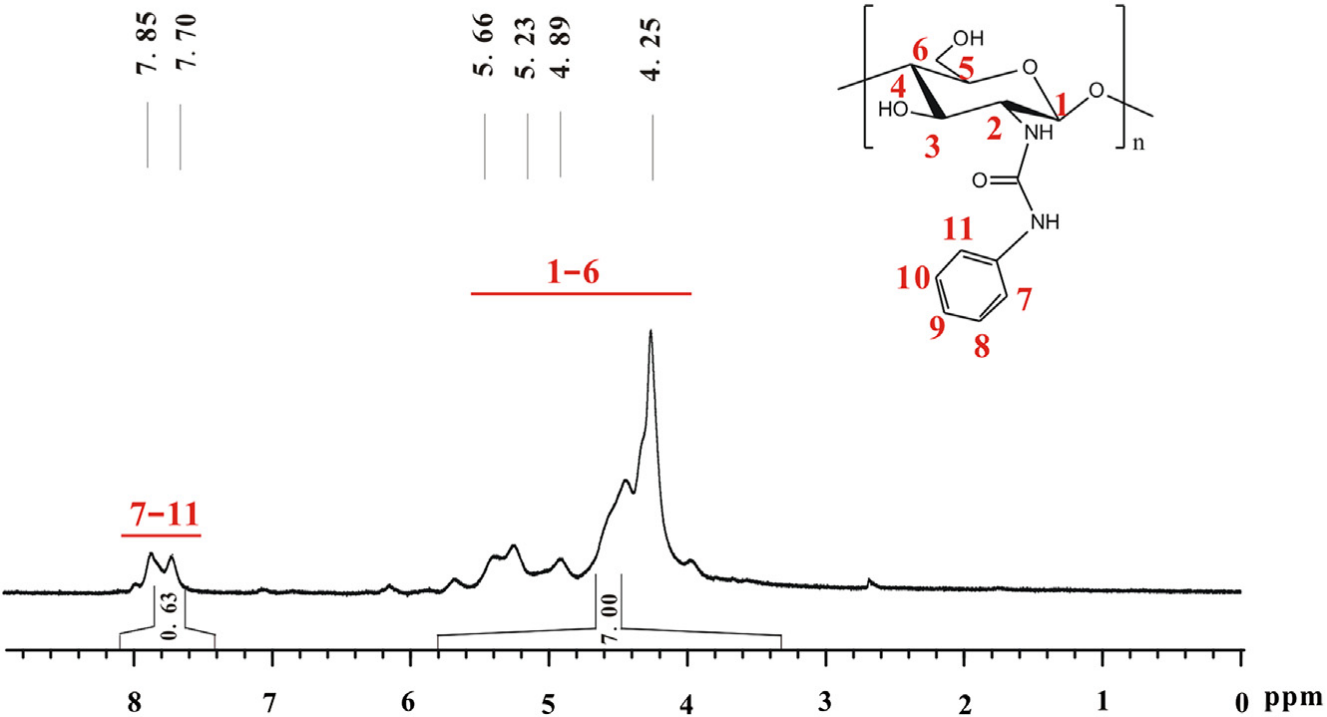
^1^H NMR spectrum of **e** (TFA-D, 298 K, 400 MHz).

The total integral of seven hydrogens on glucose skeleton of chitosan is designated as 7.00 (Fig. 9). The *DS* of phenyl group on **e** equals (*A*/5)×100%. *A* refers to total integral of the peaks at 7.85 and 7.70, in which there are five hydrogens. *DS*=(0.63/5)×100%≈13%.

**Fig. 10:**
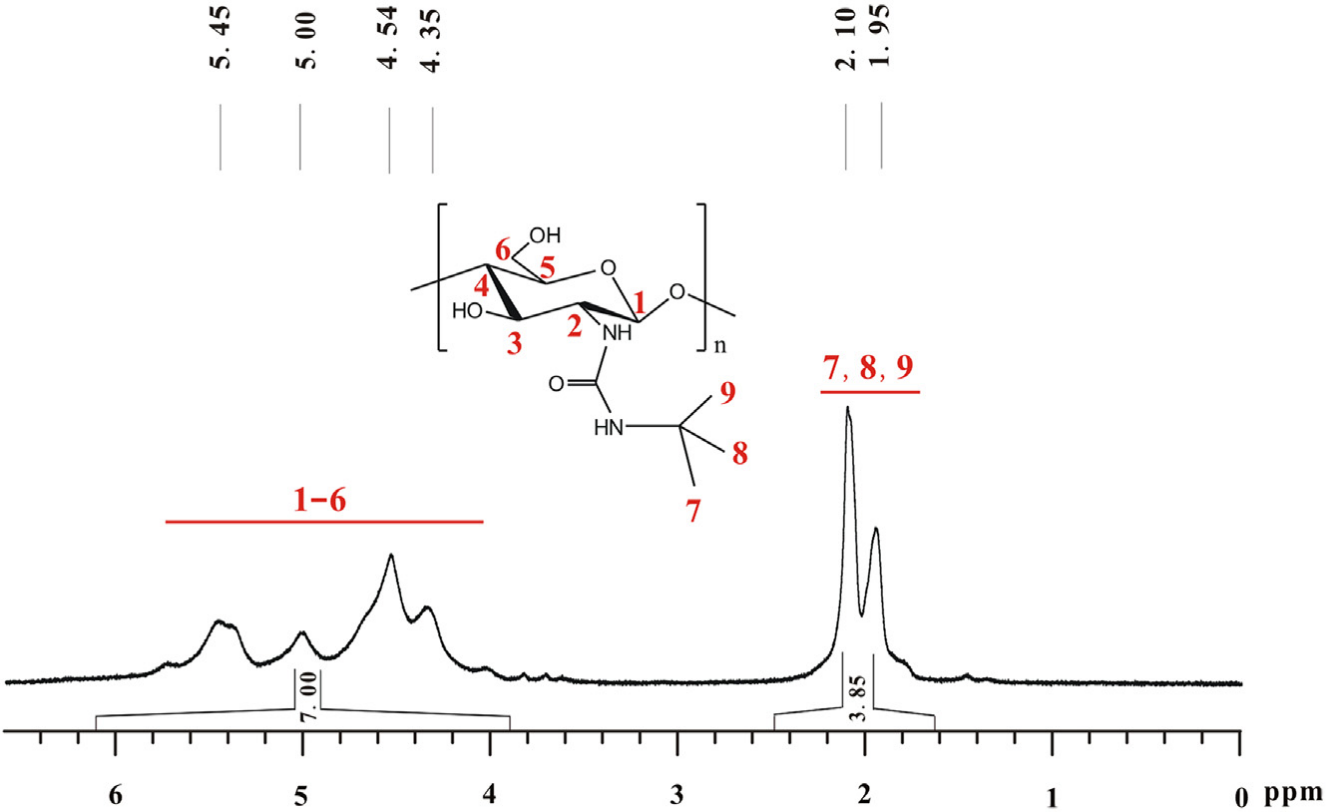
^1^H NMR spectrum of **f** (TFA-D, 298 K, 400 MHz).

The total integral of seven hydrogens on glucose skeleton of chitosan is designated as 7.00 (Fig. 10). The *DS* of *tert*-butyl group on **f** equals (*A*/9)×100%. *A* refers to total integral of the peaks at 2.10 and 1.95, in which there are nine hydrogens. *DS*=(3.85/9)×100%≈43%.

**Fig. 11:**
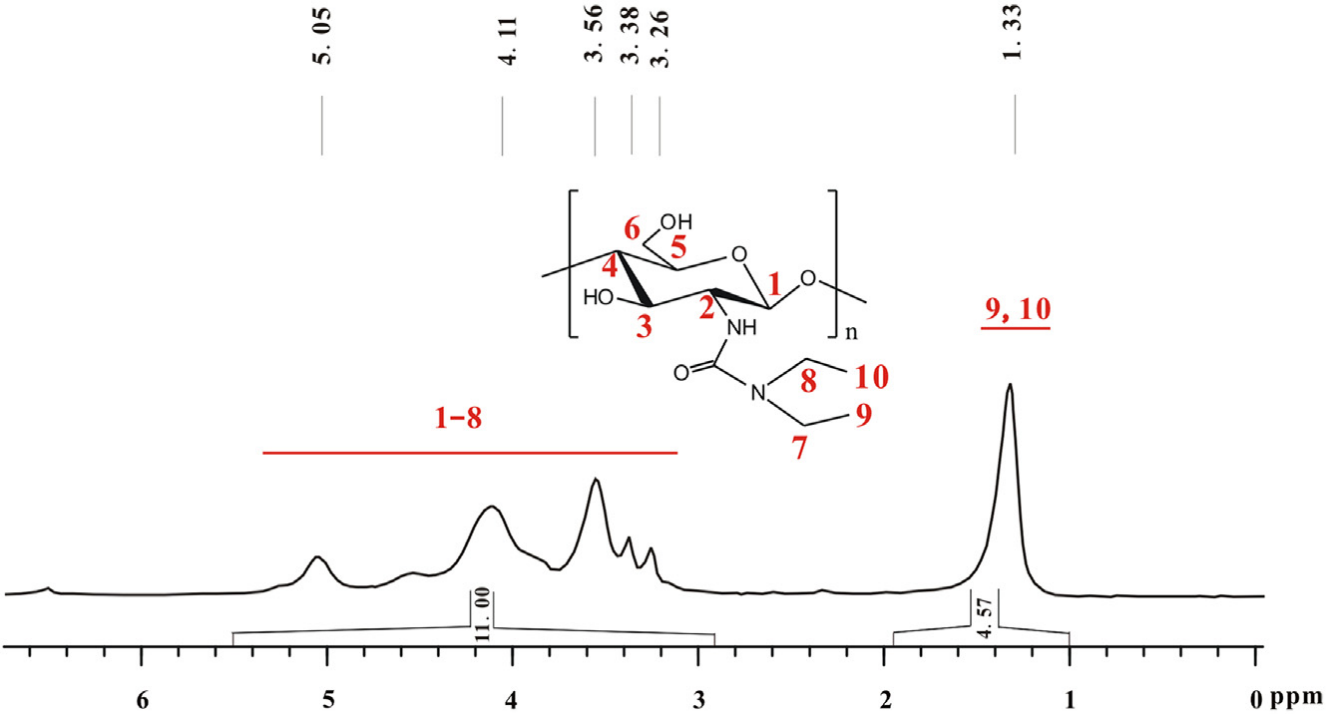
^1^H NMR spectrum of **g** (TFA-D, 298 K, 400 MHz).

The peak of four hydrogens in two ethyl groups overlapped with that of seven hydrogens on glucose skeleton of chitosan, and the total integral of these eleven hydrogens is assumed as 11.00 (Fig. 11). The *DS* of *N*,*N*-diethylamido group on **g** is calculated by the equation of (7+4*DS*)/6*DS*=11.00/4.57. Therefore, *DS*=67%.
